# The knowledge about long-term consequences of preterm birth among health professionals, educational professionals, and parents in Slovenia

**DOI:** 10.3325/cmj.2024.65.76

**Published:** 2024-04

**Authors:** Kaja Hacin Beyazoglu, Darja Paro-Panjan, Breda Šušteršič, Jana Kodrič

**Affiliations:** 1 Department of Psychology, Faculty of Arts, University of Ljubljana, Ljubljana, Slovenia; 2 Department of Neonatology, Division of Paediatrics, University Medical Centre Ljubljana, Ljubljana, Slovenia; 3 Department of Paediatrics, Faculty of medicine, University of Ljubljana, Ljubljana Slovenia; 4 Developmental Department, Health Centre Domžale, Domžale, Slovenia; 5 Child Psychiatry Unit, Division of Paediatrics, University Medical Centre Ljubljana, Ljubljana, Slovenia

## Abstract

**Aim:**

To assess the knowledge about the long-term consequences of preterm birth and the need for training and information among various professionals working with preterm children and parents of preterm children.

**Methods:**

In February and March 2018, physicians, psychologists, special education needs teachers, teachers, preschool teachers, and parents (N = 488) filled in the Preterm Birth-Knowledge Scale and a survey regarding their perceptions and attitudes toward working with preterm children.

**Results:**

Physicians and psychologists were most knowledgeable among the groups about the long-term consequences of preterm birth. Teachers, preschool teachers, and parents had significantly lower knowledge (*F* = 23.18, *P* < 0.001). The majority of professionals indicated that they did not feel adequately equipped to support the learning and development of preterm children and that they had not received sufficient training in this area. More than half indicated that they had received no formal training. In general, the participants tended to underestimate the long-term problems of preterm children.

**Conclusion:**

The findings underscore the importance of integrating the issue of the long-term outcomes of preterm birth and working with preterm children into formal education, and in other forms of educational activities.

Preterm birth (birth before 37 weeks of gestation) is associated with adverse neurodevelopmental outcomes in infancy and beyond. Extremely preterm infants (born before 28 weeks of gestation) and very preterm infants (born before 32 weeks of gestation) are at a greater risk for health, sensory, motor, cognitive, emotional, behavioral, and academic problems later in life (1). The preterm population more frequently suffers from general cognitive deficits and deficits in processing speed, attention, visual and perceptual skills, memory and learning, language, and executive functions (2). Cognitive deficits are relatively stable from the second year of life into adulthood (3-6). However, the majority of very preterm infants develop normally later in life, without significant neurodevelopmental problems (1,7).

In Slovenia, the preterm birth rate in 2020 was 7.3% (1.4% of children were born extremely preterm and very preterm, <32 weeks of gestation) (8). In a small sample of preterm children, the proportion of children with special education needs (SEN) was higher (33%) than in the general population (6).

Professionals who work with preterm children should be well educated about the problems that these children may have. Neonatologists’ knowledge of the long-term consequences of preterm birth is relatively good (9). However, the knowledge of teachers, psychologists, and nurses seems to be weaker (10-12). The areas about which they were least knowledgeable were preterm children’s mathematical difficulties, peer relationship problems, and inattention (9,11). Greater knowledge was exhibited by professionals who taught in SEN schools or had an SEN role and more than 16 years of teaching experience. Only a small minority of them felt adequately trained to support preterm children in schools (11). Although US teachers’ knowledge of the consequences of preterm birth was similarly low, those who had professional experience with a preterm child scored higher on a knowledge test than those without such experience (10). In addition, pediatric nurses showed lower knowledge than neonatologists and comparable knowledge to that of educational psychologists and teachers. At the same time, the number of years in the profession did not affect knowledge, but the professionals’ perceived level of preparation for the care of preterm children did (12).

One of the most important aspects when it comes to adequately supporting the development and learning of preterm children is the collaboration of individuals with different educational and professional backgrounds. Parents of preterm children also play an important role in this collaboration. They often report feeling unprepared to address the needs of their children (13). However, no studies to date have examined parents’ actual knowledge of long-term outcomes. Such findings could be important for planning interventions to promote the children’s development and learning. In addition, parents also felt that educators lacked understanding and awareness of their children’s special needs (14), which underscores the importance of consensus among all those working with preterm children.

Previous studies have already assessed the knowledge of some professional groups. However, we wanted to include a broader range of individuals involved with preterm children (eg, various professionals and parents). Previous studies also did not compare the knowledge of individuals with the same formal education but working in different settings. The aim of this study was to assess the knowledge about the long-term consequences of preterm birth, and the need for training and information among physicians, psychologists, and other professionals working with preterm children, as well as the parents of these children. In addition, we aimed to compare the knowledge of individuals with the same formal education but working in different settings (eg, health care institutions, educational institutions).

## Method

### Procedure and participants

In February and March 2018, an invitation email containing information about the study and the hyperlink to the web survey was sent to school and clinical psychologists through their professional associations (Section of Psychologists in Education of the Slovenian Association of Psychologists, Slovenian Chamber of Clinical Psychologists), to physicians working in neonatology departments and developmental departments in Slovenia, and to Pika Educational Centre for the training of teachers and other professionals working with children with SEN. Additional invitation emails were sent to school and preschool principals who participated in our previous studies, asking them to inform their teachers about the survey. Participants were asked to forward the invitation letter with the survey link to their colleagues and the parents of preterm children they knew, and invite them to participate. All participants completed the questionnaire anonymously.

The participants were asked if they were responding as professionals or parents, as well as about their sex and age group. Psychologists and SEN teachers were asked if they worked in a health care or educational setting.

### Measures

The Slovenian adaptation of the Preterm Birth-Knowledge Scale (11) was used. It includes 33 evidence-based statements with three forced-choice response options (true, false, don’t know) scored as 1 for correct answers, and 0 for incorrect and “don’t know” answers. The Preterm Birth-Knowledge Scale has already demonstrated good psychometric properties (9,11).

Modified answer options (“true” and “false” answers with the option to skip answers) were used. We removed the “don’t know” option that is present in the original questionnaire because this option may introduce construct-irrelevant variance into the knowledge measurement, as the final score could be influenced by various types of bias (eg, risk-taking tendency) and not only by knowledge level (15,16). Correct answers were scored 1 point, incorrect and missing answers 0 points, with the maximum score of 33 points. The Slovenian version of the scale had not been previously validated. The terms “very” and “extremely preterm” were defined in the introduction.

Professionals also filled in a survey consisting of the following questions: 1) How likely are you to come into professional contact with a very preterm child?; 2) Who is responsible for teaching very preterm children in schools (SEN teachers or a regular teacher)?; 3) Would the disclosure of the child’s preterm birth status be beneficial to the child?; 4) Could the disclosure lead to problems because of the negative effect of labeling?; 5) Do you feel adequately equipped to support the learning and development of preterm children?; 6) Have you received sufficient training for this?; and 7) Would you like to know more about strategies to support the learning and development of preterm children? These answers were recorded on a five-point Likert scale (strongly disagree; disagree; neither agree nor disagree; agree; strongly agree).

The parents were asked about their opinion on the responsibility for teaching a preterm child and the possible positive or negative effects of disclosing the child’s preterm birth status (the answer possibilities were the same as for psychologists and educational professionals).

The professionals were asked whether they had received any formal training on outcomes for preterm children (response categories for “yes” were professional training, conference, information sheet, seminar, or other) and in what way they would like to receive information about supporting preterm children’s learning and development (response categories were information sheets, internet resources, seminar, special module as part of standard professional training, “I don’t need any more information,” and other resources).

The parents were asked if they had received enough information from professionals about children’s development and learning (yes or no) and in what way they would like to receive this information (information sheets, internet resources, lectures, or workshops, “I don’t want more information,” and other resources).

### Statistical analysis

The normality of distribution was tested with graphical methods (histogram, boxplot), the Shapiro-Wilk test, and the coefficients of skewness and kurtosis of the distribution. Differences between participant groups were evaluated with a one-way ANOVA and a Tukey HSD *post-hoc* test. Differences between health and education professionals were assessed with an independent-samples *t* test. The statistical significance level was 0.05. The statistical analysis was performed with IBM SPSS for Windows, version 22.0 (IBM Corp., Armonk, NY, USA).

## Results

Of the 866 participants who filled in the survey, 488 (56.4%) who answered all 33 questions were included in the analysis. Most of them were teachers (n = 131; 26.8%), followed by preschool teachers (n = 89; 18.2%), parents (n = 85; 17.4%), psychologists (n = 81; 16.6%), SEN teachers (n = 67; 13.7%), and physicians (n = 35; 7.2%). The demographic characteristics of the sample are shown in Table 1.

**Table 1:** Respondents’ demographic characteristics

N (%)	Physicians	Psychologists	SEN teachers	Teachers	Preschool teachers	Parents
All	35	81	67	131	89	86
Sex^†^						
male	4 (11.4)	0 (0.0)	0 (0.0)	10 (7.6)	1 (1.1)	2 (2.3)
female	31 (88.6)	80 (98.8)	66 (98.5)	121 (92.4)	87 (97.8)	83 (96.5)
Age (years)						
<30	9 (25.7)	24 (29.6)	10 (14.9)	6 (4.6)	9 (10.1)	11 (12.8)
31-40	15 (42.9)	25 (30.9)	16 (23.9)	37 (28.2)	37 (41.6)	57 (66.3)
41-50	2 (5.7)	20 (24.7)	25 (37.3)	47 (35.9)	23 (25.8)	13 (15.1)
51-60	6 (17.1)	7 (8.6)	15 (22.4)	39 (29.8)	20 (22.5)	3 (3.5)
>60	3 (8.6)	5 (6.2)	1 (1.5)	2 (1.5)	0 (0.0)	1 (1.2)
Work setting						
medical		27 (33.3)	8 (11.9)			
educational		34 (42.0)	46 (68.7)			
other		20 (24.7)	13 (19.4)			

*SEN – special education needs.

†Data on sex are missing for one of the respondents in the groups of psychologists, special education teachers, preschool teachers, and parents.

Compared with the national data on sex ratios in education (17), there were slightly fewer men in our sample (national data for preschool teachers = 2.7% of men; school teachers = 11.7% of men), whereas national data for other professions are not publicly available.

All of the physicians were pediatricians or pediatric residents. Thirty-four psychologists (42%) and 46 (68.6%) SEN teachers worked in public educational settings, while 27 (33.3%) psychologists and 8 (11.9%) SEN teachers worked in public medical settings. The rest worked in other institutions.

### Knowledge of preterm birth

The average knowledge score for all respondents was 20.12 (standard deviation 3.44). One-way ANOVA revealed significant differences in knowledge scores between groups (*F* = 23.18, *P* = <0.001). Physicians and psychologists had significantly higher scores than other groups. SEN teachers had significantly lower scores than physicians and psychologists, but had significantly higher scores than other groups. Teachers, preschool teachers, and parents had the lowest scores, and the differences between them were not significant (Table 2).

**Table 2:** Average knowledge scores for each group of respondents

Group	Mean	Standard deviation	ANOVA (Tukey HSD test)	Mean difference	Standard error	*p*
Physicians	22.86	3.17	psychologists	0.413	0.627	0.986
			SEN teachers	2.036	0.647	0.021
			teachers	3.544	0.590	<0.001
			PT	4.172	0.619	<0.001
			parents	3.892	0.623	<0.001
Psychologists	22.44	2.85	SEN teachers	1.624	0.512	0.020
			teachers	3.131	0.438	<0.001
			PT	3.759	0.476	<0.001
			parents	3.480	0.482	<0.001
SEN teachers	20.82	3.45	teachers	1.508	0.466	0.016
			PT	2.136	0.502	<0.001
			parents	1.856	0.507	0.004
Teachers	19.31	3.18	PT	0.628	0.426	0.682
			parents	0.348	0.432	0.966
PT	18.69	2.94	parents	–0.279	0.470	0.991
Parents	18.96	3.06				

*Abbreviations: SEN – special education needs; PT – preschool teachers.

Psychologists working in health care institutions had significantly higher scores (23.48 ± 2.56) than psychologists working in educational institutions (21.62 ± 3.19) (*t* = 2.47, *P* = 0.017). Similarly, SEN teachers working in health institutions scored higher (23.00 ± 2.39) than those working in educational institutions (20.39 ± 3.76), but the difference was not significant (*t* = 1.89, *P* = 0.064).

### Specific areas of knowledge

**Figure 1:**
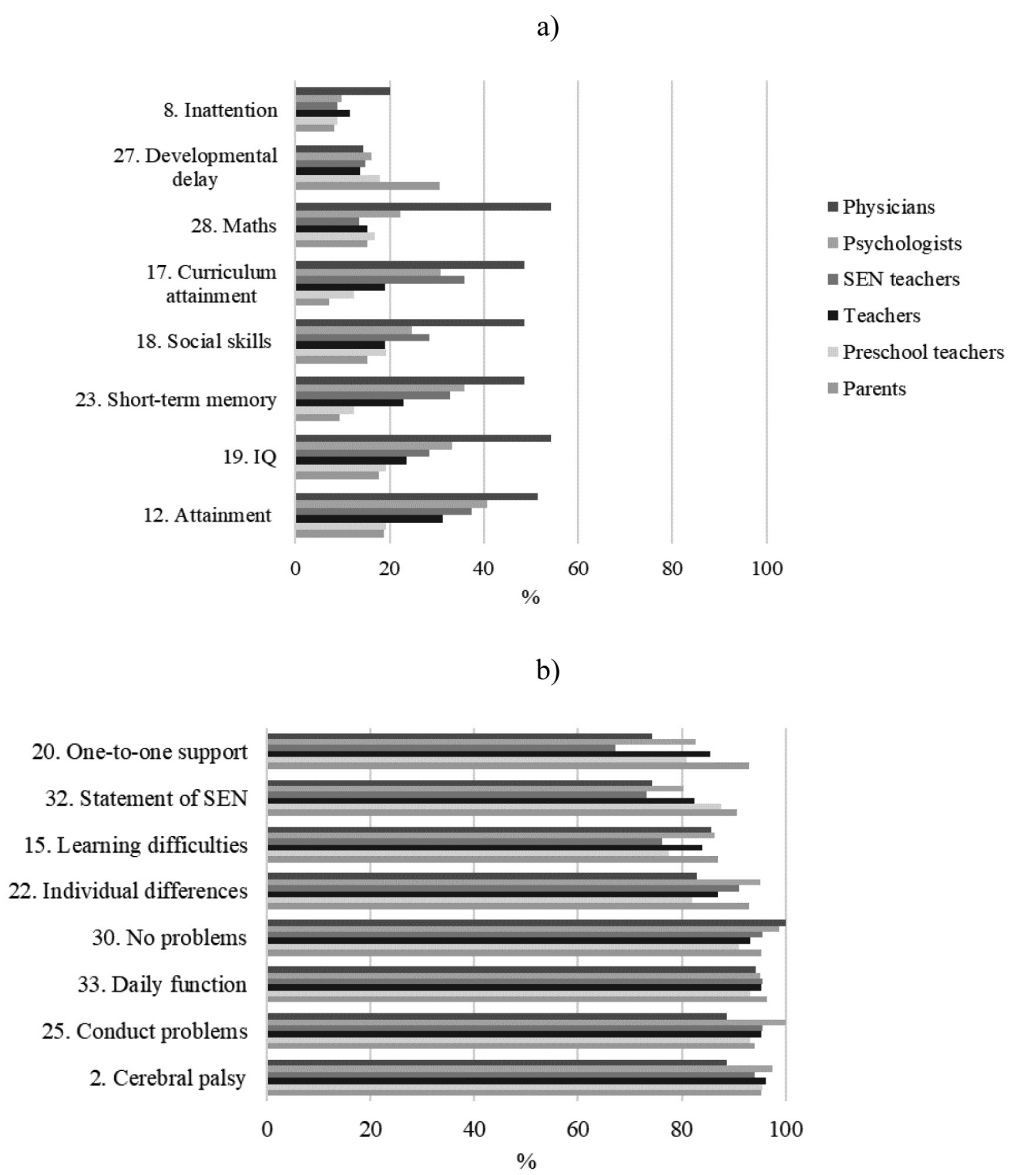
The items with the fewest (**A**) and the most (**B**) correct answers. SEN – special education needs.

Next, we determined the specific areas of knowledge of the different professional groups by examining the eight items with the fewest correct answers (bottom 25% items) and the eight items with the most correct answers (top 25% items) (Figure 1).

In general, all groups were fairly congruent in terms of the items with the lowest scores. The majority of the items in the bottom 25% were items that described some problems of preterm children and were true, or items that stated that preterm children were in some aspects the same as their peers and were false. As regards the highest scoring items, most of these described some of the problems of preterm children, but were in fact false. The exception was item 30, which states that some children born very preterm will not have problems later in life, and is true.

We also assessed the differences between the groups in relation to specific areas of knowledge. For example, among physicians, psychologists, and SEN teachers, items 1 (gradient in outcome), 6 (attention deficit/hyperactivity disorder), and 7 (attainment in non-impaired) were in the top 25%. Interestingly, although physicians generally scored highest, they scored lower than other groups on items 10 (severe disability) and 14 (hyperactivity/impulsivity). The groups showed the greatest differences regarding item 14, with teachers, preschool teachers, and parents scoring higher on this item than other groups. Parents also scored higher than other groups on items 31 (reading) and 32 (statement of SEN).

### Training and information needs

**Figure 2:**
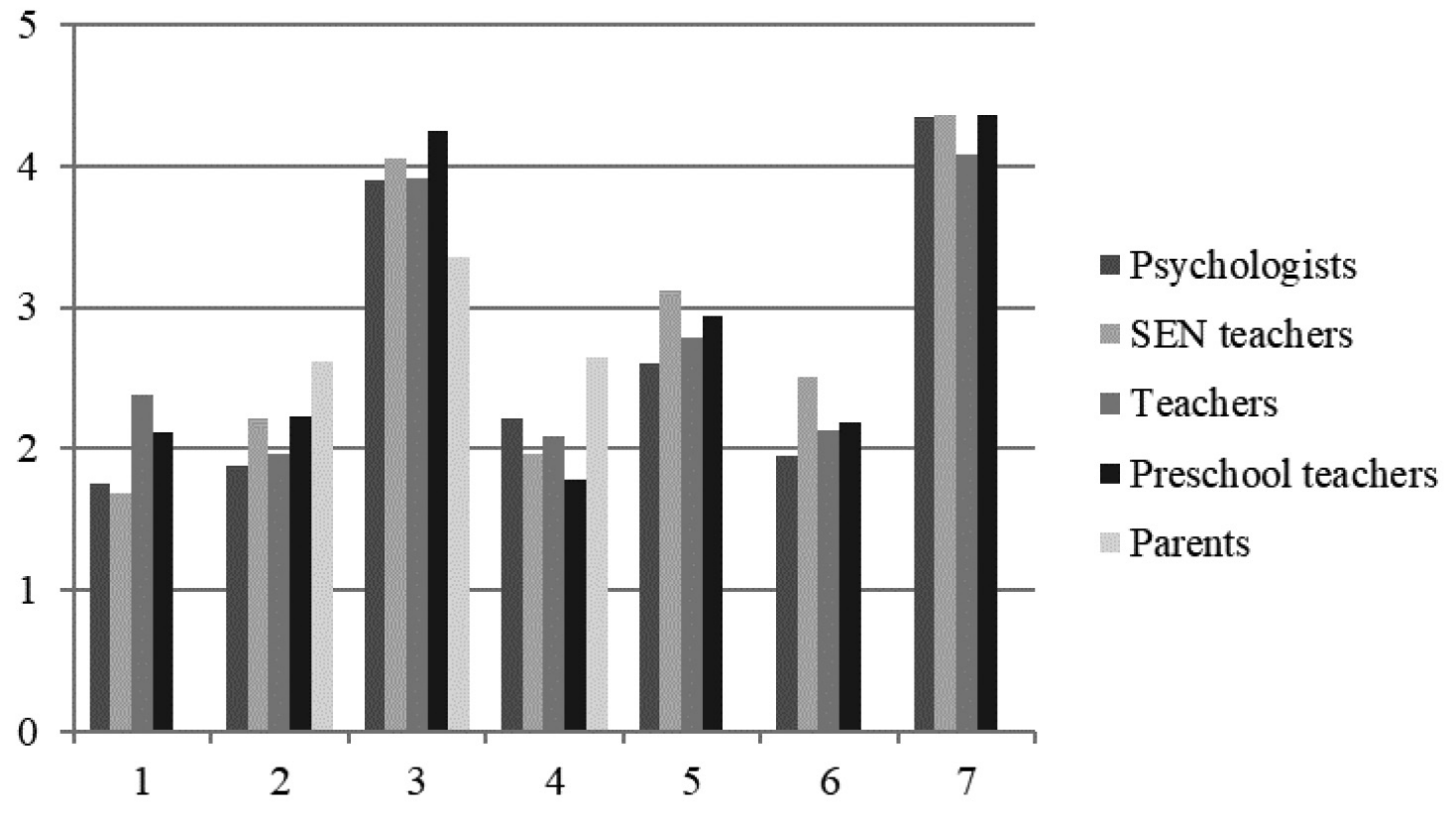
Respondents’ opinions on issues related to the education of preterm children (1-4) and on training received (5-7). The x-axis shows the ordinal numbers of the statements for which the participants indicated their agreement on a five-point scale. The y-axis shows the average score for each occupational group for each of the items assessed. SEN - special education needs. 1 = I am unlikely to come into professional contact with a child born very preterm; 2 = Educational management of very preterm children is the job of the SEN coordinator, not the class teacher; 3 = Disclosing a child’s preterm birth status to the school would be beneficial for the child; 4 = Disclosing a child’s preterm birth status would lead to problems because of the negative effect of labeling; 5 = I feel adequately equipped to support the learning and development of preterm children; 6 = I have received sufficient training in how to support the learning and development preterm children; 7 = I would like to know more about strategies I can use to help support the learning and development of preterm children.

All the professional groups agreed that they would like to know more about strategies to support the learning and development of preterm children (Figure 2). They felt they had not received sufficient training in this area. At the same time, they felt that disclosing the child’s preterm birth status to the school would be beneficial to the child and would not lead to negative labeling. Interestingly, parents were less likely to see the benefits of disclosing their child’s preterm status to the school and more likely to be concerned about the negative effect of labeling.

The majority of respondents in all professional groups indicated that they had not received any formal training on the topic of preterm birth (Table 3). The exception were physicians, 38.3% of whom reported that they had not received any formal training. On the other hand, the most common method of formal training remained professional training or conferences. Teachers reported having received the least formal training, regardless of the method of training used.

**Table 3:** The method of formal training on preterm birth received by different professional groups

	Physicians (%)	Psychologists (%)	SEN* teachers (%)	Teachers (%)	Preschool teachers (%)
None	38.3	54.3	59.7	72.5	65.2
As a part of professional training	14.9	12.3	25.4	5.3	18.0
At a conference	21.3	13.6	11.9	5.3	1.1
Via an information sheet	10.6	9.9	14.9	3.1	4.5
At a seminar	10.6	3.7	9.0	1.5	0.0

*SEN – special education needs.

**Figure 3:**
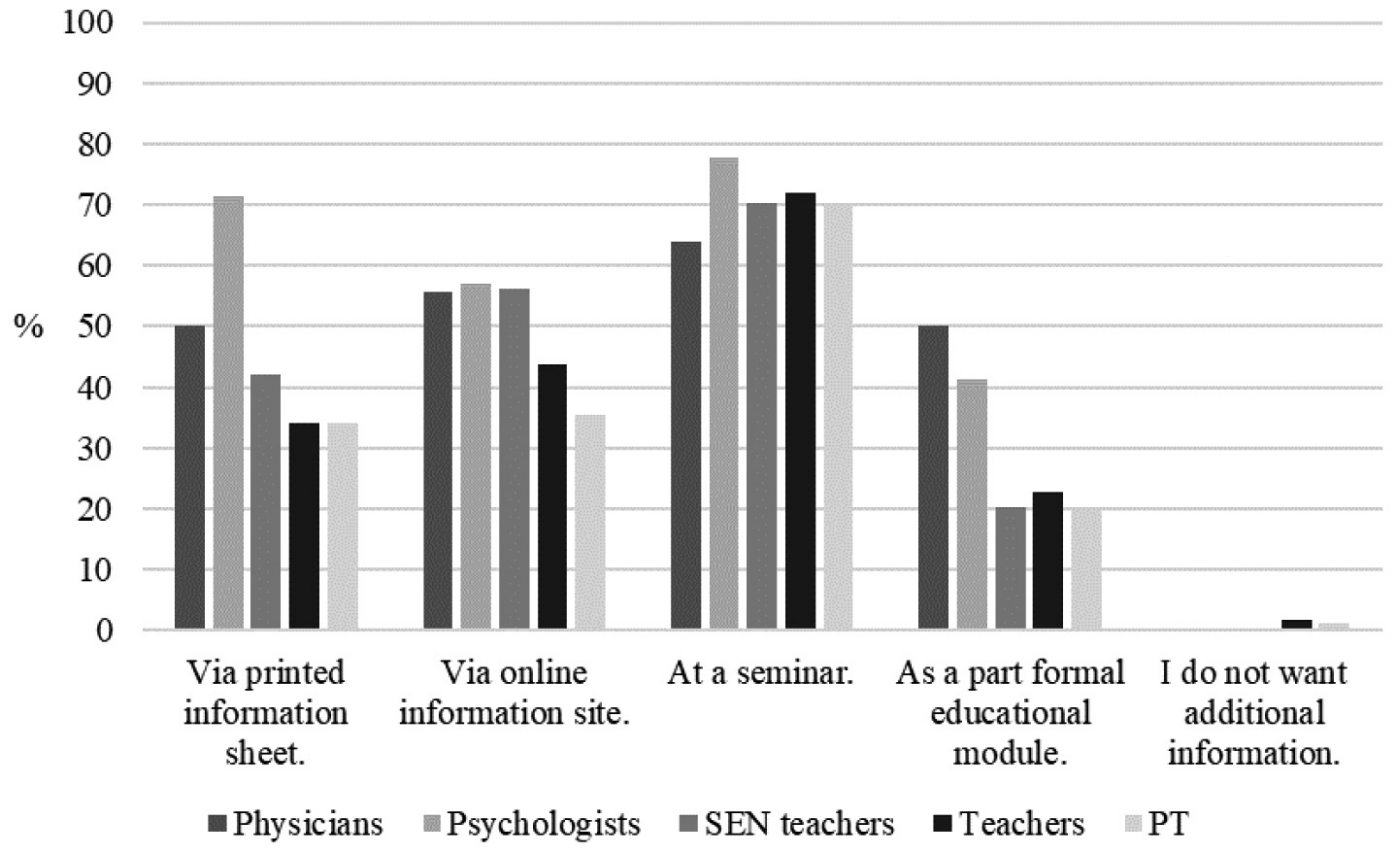
Professionals’ responses on the preferred way to receive additional information about preterm children. SEN – special education needs.

The majority of professionals indicated that they would like to receive additional information at an educational seminar, followed by an online information site. Almost none of the participants answered that they did not want any additional information (Figure 3).

Overall, 61.9% of parents felt that they had received enough information from professionals about the development and learning of preterm children. However, only 10.7% indicated that they did not want more information or other resources. Parents preferred lectures or workshops (60.7%) and internet resources (56.0%) to obtain additional information, followed by information sheets (39.3%).

## Discussion

In this study, physicians and psychologists showed the best knowledge about the consequences of preterm birth, followed by SEN teachers, whereas teachers, preschool teachers, and parents had lower knowledge. These results are consistent with the findings of previous studies (9-12).

Similar to previous studies (9,11), physicians had the best knowledge about the long-term consequences of preterm birth, whereas the knowledge of teachers and preschool teachers was weaker. However, psychologists’ knowledge was higher compared with other groups than in previous studies (9,11). This may partly indicate the importance of a specific educational program. Accordingly, the absolute scores of teachers and preschool teachers in our study were also higher than in previous studies. At the same time, the comparison of the absolute scores between our study and previous studies was limited because we used different response options, which could have affected participants’ absolute scores on the test.

In addition, the finding that teachers, preschool teachers, and parents had the least knowledge about the consequences of preterm birth is important because all three groups spend the most time with these children on a daily basis, and therefore may be the first to recognize the children’s problems.

At the same time, the level of knowledge is consistent with the professionals’ self-reports of formal training on the consequences of preterm birth. More than two-thirds of teachers and preschool teachers reported having received no formal training. Combined with low knowledge scores, this underscores the importance of formal training to ensure that professionals have adequate knowledge of various issues that these children may face. Even a month-long training course based on e-learning resources can significantly improve teachers’ knowledge of preterm birth (18).

In addition to the influence of formal education, our study showed that the work environment can also affect professionals’ knowledge. Both psychologists and SEN teachers working in health care settings scored higher on the knowledge test than their counterparts working in educational settings. This is consistent with the finding that professionals experienced in the work with preterm children perform better on the knowledge test (10), as professionals working in a health setting are more likely to interact with preterm children who have developmental problems.

The analysis of the items with the most and least correct answers showed that the majority of participants in our study tended to underestimate the problems of preterm children. The items with the fewest correct answers were the true items that mentioned various problems of preterm children and the false items that stated that children did not have specific problems. In other words, participants believed that preterm children were similar to their peers in certain aspects, when in fact they were more likely to have problems in that area. Similarly, the items with the most correct answers were those that included false statements. In summary, the majority of participants in our study believed that prematurely born children did not have specific problems and were comparable to their peers. These beliefs probably stem from the fact that most premature babies do not have problems later in life, which is consistent with other research (1,7). Since only a fraction of preterm children have problems, participants might have been less likely to encounter these children outside of health care settings. For example, even if a teacher encounters a child with developmental problems in the classroom, he or she may not necessarily know that the child is preterm. Combined with a lack of formal education, this could lead to a lack of awareness that preterm birth can be a risk factor for developmental problems. This also highlights the shortcomings of developing professional knowledge and skills based solely on everyday work experiences. It also emphasizes the importance of integrating this content into various forms of training.

Interestingly, for two items (“Only a small proportion of children born very preterm will have severe disabilities later in life.” and “Children born very preterm are likely to be hyperactive and disruptive in the classroom.”), physicians overestimated the problems of preterm born children. Similar to the above, this bias could also arise from the professional experience of physicians. In their work, physicians are probably more likely than other professionals to encounter preterm children with developmental problems, which could give them the subjective impression that a higher proportion of these children have severe disabilities.

None of the groups of professionals felt well equipped to support preterm children, and they all felt that they had not been adequately trained in this respect. This finding underscores the importance of additional training and education for professionals who work with preterm children. In one study, educators reported being less confident in their knowledge about developmental consequences of preterm birth than in their knowledge about other neurodevelopmental disorders (13).

Professionals and parents may have different perspectives and attitudes. One such issue that emerged from our study is the disclosure of a child’s preterm status to the school. All professionals in our study seemed to agree that this disclosure would be beneficial and would not have the negative effect of labeling. However, the parents were less likely to agree that the disclosure of their child’s preterm status would be beneficial, and more likely to agree that it could lead to problems.

In summary, there were some important differences between various groups of professionals in terms of their knowledge about preterm children. These differences could be due to the lack of formal training on the subject and experience working with preterm children. Professionals often feel ill-equipped to support preterm children and would like additional information on this topic. In fact, the majority of the professionals would like to attend an educational seminar on supporting preterm children. At the same time, information sheets and websites seemed to be a good option for them.

The study has some limitations. The comparability of the absolute scores of the different groups discussed in published studies is somewhat limited because of the different response options in the knowledge test. In addition, the sample might not have been representative in terms of sex ratio. In the future, the knowledge of the different professional groups should be assessed using a large and representative sample.

Our results indicate the need for additional training in the form of online seminars or courses, as they are effective methods for providing knowledge about preterm children’s development (18). The majority of parents would like to receive additional information on preterm children’s development and learning, either in the form of lectures or workshops, or from internet sources.

In conclusion, professionals working in health care settings have more knowledge about preterm children than those working in school settings. This indicates that formal education and everyday experience with preterm children with adverse outcomes play an important role in this type of knowledge. The results of our study suggest that parents, educators, and health care professionals need to be more systematically informed about the development and learning of preterm children, and this may be done through online information or training.
